# Telemedicine and Cardiology in the Elderly in France: Inventory of Experiments

**DOI:** 10.1155/2019/2102156

**Published:** 2019-01-21

**Authors:** A. A. Zulfiqar, A. Hajjam, B. Gény, S. Talha, M. Hajjam, J. Hajjam, S. Ervé, E. Andrès

**Affiliations:** ^1^Department of Geriatrics, University Hospital of Rouen, France; ^2^Research Unit EA 3072 “Mitochondrie, Stress Oxydant et Protection Musculaire”, Faculté de Médecine de Strasbourg, Université de Strasbourg (UdS), 4 rue Kirschleger, 67091 Strasbourg, France; ^3^Research Unit EA 4662 “Nanomédecine, Imagerie, Thérapeutiques”, Université de Technologie de Belfort-Montbéliard (UTBM), Belfort-Montbéliard, France; ^4^Service de Physiologie et d'Explorations Fonctionnelles, Hôpitaux Universitaires de Strasbourg (HUS), 1 porte de l'Hôpital, 67091 Strasbourg Cedex, France; ^5^PREDIMED Technology, Mulhouse, France; ^6^Centre d'Expertise des Technologies de l'Information et de la Communication pour l'Autonomie (CenTich) et Mutualité Française Anjou-Mayenne (MFAM), Angers, France; ^7^Department of Internal Medicine, Diabetes, University Hospital of Strasbourg, 1 porte de l'Hôpital, 67091 Strasbourg Cedex, France

## Abstract

Telemedicine is now in vogue, allowing through computer and communication tools to be deployed in the field of health, such as cardiology, area in which it has shown interest, in international studies. As the population ages, older people are increasingly concerned with this innovative practice. We take a look at telemedicine projects in France concerning the elderly, in the field of cardiology.

## 1. Introduction

The development of information and communication technologies (ICTs) for the elderly is a promising venture. They create new opportunities to assist and care for elderly people at home or in specialized institutions, including nursing homes and hospitals. Grouped under the term* gerontechnology*, the first analyses of their contributions to this field appeared in the mid-1990s.

Cardiovascular disease, including heart failure and arrhythmia, remains significant among elderly and very elderly subjects. Indeed, heart failure affects over 10% of the population at the age of 80 and over. Furthermore, heart disease is linked to very high mortality rates, as well as to a very high rate of rehospitalization; one in two known sufferers will be rehospitalized within six months of receiving this diagnosis [1].

In this article, we have provided a review of both the literature and web concerning telemedicine projects, developed in the CHF setting. We have restricted our searches to projects and trials using nonintrusive devices. We have reported on the experience made with current telemonitoring projects in France, in the field of cardiology in the elderly. Our work focuses exclusively on telemedicine in cardiology in the elderly in France, whether on heart failure or rhythmology, contrary to a previous work done by the same team, focusing on heart failure in any subject, with telemedicine projects 1.0 and 2.0 developed around the world [2]. A similar work in terms of methodology was carried out by the same authors in the field of teledermatology focused on the elderly [3] and in the field of telemedicine projects in geriatrics [4] in France.

## 2. Method

A literature search was performed on the* PubMed *database of the* US National Library of Medicine *and on* Google Scholar*. We searched for articles published, using the following keywords or associations:* “heart failure”, “telemedicine”, “telemedicine in heart failure”, “elderly”, “arrhythmias”, “telemedicine in arrhythmias”. *The search was restricted to studies published in the era of French telemedicine projects or studies in the field of cardiology in the elderly exclusively.

## 3. Telemedicine and Heart Failure in the Elderly

The SEDIC study (*Suivi Educatif à Domicile des Patients avec Insuffisance Cardiaque *[Educational Home Monitoring of Patients with Heart Failure], from France's Basse-Normandie region) studied the suitability of a telemedicine program in the context of clinical home monitoring (*Suivi Clinique à Domicile *[SCAD]) beginning in 2007. The patient's care is organized based on the collection of data entered by the patient and sent to the educational monitoring center. This data collection corresponds to a randomized, open, multicenter prospective trial evaluating the impact of educational follow-up via telemedicine for a period of three months in patients over the age of 65 who have been hospitalized for acute heart failure (LVEF <45%). The main evaluation criterion is the number of days of hospitalization for an acute cardiac event at the one-year mark. The preliminary results after three months deal with 73 patients, of whom 35 were randomized into the conventional treatment arm and 38 into the telemedicine arm. There was no significant difference in the main evaluation criterion. At the conclusion of this study, 1040 days of hospitalization for acute heart failure were recorded. Educational telemedicine monitoring made it possible to reduce the number of days (control group: 590 days vs. telemedicine group: 450 days). Quality-of-life factors improved similarly in the two groups. A significant decrease in cardiovascular mortality was noted [5].

Several points and results are worth highlighting and discussing in view of the data from the literature. In the SEDIC study, the average age of patients was 76.8 years, higher than in other studies, such as the average age of 61 years in Tele-HF (“Telemonitoring to Improve Heart Failure Outcomes”) [6]. In terms of therapy, the patients coming out of the SEDIC study received better treatment than those in Tele-HF. In the SEDIC study, 20% of patients had one or more cardiovascular events within three months (death or rehospitalization), results similar to those of Rich et al. [7]. The results after the first three months of the SEDIC study, though not statistically significant, reinforce the notion that telemedicine is of practical interest in the follow-up care of elderly patients with heart failure.

Moreover, in this population, telemedicine may reduce mortality. In the TEN-HMS study (Trans-European Network-Home-Care Management System) [8], the patients monitored via telemedicine lost fewer days of life when compared with the patients in the conventional monitoring group. The meta-analyses are positive, with a reduction in morbidity and mortality in the telemonitored groups; these meta-analyses include those conducted by the Cochrane Group and by Inglis et al. [9].

With regard to the SEDIC study, over the course of three months of telemonitoring, the 42 patients sent out 319 alerts; only four patients sent out no alerts. The top cause for these alerts was the aggravation of respiratory symptoms, followed by weight gain. Nurses responded to 204 alerts (131 phone calls, 73 messages on tablets). The most common paramedical response was a reinforcement of therapeutic education, along with advice to consult the patient's doctor in the absence of clinical improvement. It is worth noting that the telemonitoring system suffered from the issuing of false alerts, in particular for weight gain [10]. As for the patients who did not respond to this educational telemedicine in the SEDIC study, 16 of the 45 patients in the educational telemedicine group died or were rehospitalized for acute heart failure within the year (36%). The nonresponsive patients were more often symptomatic upon enrollment in the study, with more impaired quality of life and a higher geriatric depression score. On the other hand, none of the strong prognostic markers of heart failure, such as BNP, left bundle branch block, LVEF, six- minute walk test, or hemoglobin level, stood out in univariate analysis. Thus, patients who were nonresponsive to educational telemedicine were more often symptomatic and had a higher depression score upon enrollment than the responsive patients [11].

A study by Dary P aims to use at-home telemonitoring to evaluate the idea of optimizing treatments as a possible alternative to hospitalization [12]. 29 women and 54 men were included, with an average age of 78 years, 41% with a preserved ejection fraction and 59% with an altered ejection fraction. To evaluate the benefits of short-term telemonitoring, weight, blood pressure, and electrocardiograms were collected using a take-home telemedicine kit. The telemonitoring occurred directly between the patient and the cardiologist, so the treatment could be adapted in real time. Telemonitoring is not used as an alert system but rather aims to continually adapt the treatment according to the results of measured parameters. The results show an average weight loss of 2 kg (p<0.0001), linked to a 50% increase in the dosage of diuretics. During edema flare-ups, the rate of weight loss is a prognostic tool; if slow, it can predict hospitalization. Weight is an effective warning sign and powerful educational tool: it can predict short-term developments and indicate whether a situation is getting worse. The decrease in blood pressure is limited to 6 mmHg for systolic pressure (p = 0.002) and 7 mmHg for diastolic pressure (p <0.0001), allowing an increase in inhibitors of the conversion enzyme/sartans. The heart rate changes from 87 to 73 bpm (p <0.0001), which is more important in cases of atrial fibrillation. This is the second important criterion, after weight control that predicts the risk of hospitalization. One of the benefits of this method is the option to offer an alternative to hospitalization for 31 patients (37%)—specifically, management during edema flare—ups and emergencies, thus avoiding hospitalization. During the first 30 days after leaving the hospital, 4.4% of patients died, while 5% were readmitted for recurrence. This figure rose to 20% when all causes were included. In 2003, Goldberg et al. [13] published the WHARF study, which included the largest randomized multicenter sample and compared the value of telemonitoring aftercare to that of in-person aftercare. After 6 months, there was no difference in the rate or time frame of hospital readmissions (p = 0.28), but there was a significant reduction in mortality rates (p <0.003). The Telemonitoring to Improve Heart Failure Outcomes (Tele-HF) study [6] included patients who had presented with decompensated heart failure in the last 30 days. The average age was 61. There was no notable difference between the two groups based on the criteria of mortality rate and hospitalization for any cause, nor on the secondary criteria of death, hospital readmission, and length of hospital stay.

In this context, Andrès et al. developed a telemedicine project called E-care, dedicated to “the early detection of risk situations in heart failure patients” (PIA, 2014) [14]. This detection is based on a web-connected touch-screen tablet; nonintrusive sensors (arterial pressure, heart rate, oxygen saturation, and weight); questionnaires; and artificial intelligence.

The E-care telemonitoring platform was rolled out with patients in the context of an experiment directed by the University Hospitals of Strasbourg (Hôpitaux Universitaires de Strasbourg, HUS, Strasbourg).

During this experiment, the E-care telemonitoring platform was used daily by patients and by healthcare professionals according to a set usage protocol tailored to each patient. The average age of these patients was 72 years, with a male/female ratio of 0.7. These patients present with multiple conditions, with a Charlson Comorbidity Index of 4.1. Among the most frequent conditions were heart failure in over 60% of these individuals; anemia in over 40%; atrial fibrillation in 30%; type 2 diabetes in 30%; and chronic obstructive pulmonary disease (COPD) in 30%.

More than 1,500 measurements were taken from these 175 patients, causing the E-care system to generate 700 “alerts” regarding 68 patients. During the monitoring period, 107 individuals (61.1%) had no “alerts.” The follow-up study on those 107 patients shows that they presented no clinical health event that could result in hospitalization. An analysis of the alerts shows that the E-care platform makes it possible to automatically and nonintrusively detect a decline in the patient's health status, in particular cardiac decompensation. Indeed, it is in this last situation that the system offers the best sensitivity (Se) and specificity (Spe) values, as well as positive and negative predictive values (PPV and NPV): 100%, 72%, 90%, and 100%, respectively.

The E-care project is, to our knowledge, the first such program rolled out for heart failure patients who are elderly (average age: 72), have multiple medical conditions, and belong to the geriatric population (Charlson Comorbidity Index: 4.1), using a telemedicine solution for early detection of situations that could lead to acute decompensation. In this sense, we are in the realm of predictive medicine, which, in the context of the E-care platform, aims to be increasingly personalized, which is to say, adapted to the phenotype of each patient. For the practitioner, this means that the E-care telemonitoring platform makes it possible to detect 100% of cardiac decompensations and that three-quarters of alerts refer to such cases. Only 10% of the alerts lack a direct relationship to heart failure.

To date, this is the first time that such a communicative, “smart” system has been built upon new technological tools, anticipating Telemedicine 2.0 solutions. All of the patients, including the most fragile ones among them, and the healthcare professionals used the E-care system without difficulty through the end of the experiment. During the experiment on nonautonomous patients, the system was used by a nurse, in addition to his/her other duties (washing, injection of various medications, etc.) or by the patient's family, friends, or caregivers. It should be noted that the tools and the system were tested and developed in advance by patients and professionals at the National Information Technology Center for Autonomy (Centre d'Expertise National des Technologies de l'Information et de la Communication pour l'Autonomie, “Centich,” Angers). As in this experiment, age did not appear to be a limiting factor in acquiring and using these new technologies.

Other representative telemedicine projects currently in development in France in the area of heart failure include the following:

- PIMPS (Plateforme Interactive Médecins Patients Santé, Doctor/Patient Health Interactive Platform) is a platform intended for everyone involved with heart failure, from the patient to the healthcare professional. The primary objective of this project, led by Professor Jourdain, is to demonstrate the impact of a telemonitoring program on heart failure patients, including training for professionals and reinforcement of patient education, as well as telemonitoring and therapeutic follow-up care. Moreover, it is based on home measurement of a biomarker, which is what makes it innovative. The study model involves a group of 330 heart failure patients, randomized into three groups: arm 1, including 110 patients with visits to a cardiologist every three months; arm 2, with 110 patients followed through supervised telemonitoring and regularly scheduled care; and arm 3, with 110 who enjoyed interactive follow-up through supervised telemonitoring, regularly scheduled care, and a BNP monitor [15]. The results of this study are due to be announced shortly.
- OSICAT (Optimisation de la Surveillance Ambulatoire des Insuffisants Cardiaques par Télécardiologie, Optimization of Ambulatory Monitoring of Heart Failure Patients through Telecardiology), based at 12 local university health centers, is coordinated by CHU Toulouse. The OSICAT study is a telemedicine program launched by CHU Toulouse (Professors Pathak and Galinier), with the goal of using telecardiology to optimize the ambulatory monitoring of heart failure patients. It relies upon a telemedicine association, therapeutic education, and a phone-based platform staffed with specialized nurses. Its primary objective is to evaluate the impact of a telecardiology program on morbidity and mortality in heart failure patients. The study model involves a randomized, open, controlled clinical trial with two parallel arms involving 435 patients. Among the enrollment criteria, the trial accepted heart failure patients, both men and women, over 18 years of age, who had been hospitalized in the previous 12 months for cardiac decompensation. This study, therefore, was not limited to geriatric patients. The telecardiology program includes a daily weight measurement, daily questions about symptoms associated with heart failure, and regular phone calls made by nurses. All-cause mortality and hospitalizations were evaluated after 6, 12, and 18 months in the two groups. The study will produce a medico-economic analysis [16].

The patients in the control group were treated conventionally: released to their homes with follow-up consultations with their general practitioner or their referring cardiologist. As for the patients in the telemonitoring group, within a week of their baseline visit, the materials required for their monitoring were delivered to them at home, including a connected electronic scale and an electronic unit allowing them to respond to the questionnaire evaluating the course of their symptoms. The patients in the telemonitoring group also had the opportunity to speak with nurses over the phone to ensure their full understanding of their disease and treatment and to guide them in the daily management of their disease. The results of this study will be made public soon.

- MEDICA (Monitorage Electronique à Domicile de l'Insuffisance Cardiaque Chronique, Electronic Home Monitoring of Chronic Heart Failure) is supported by Reunica Domicile and GMC-Solutions Santé.
- Cardiauvergne is handling follow-up and coordinated care for 2000 heart failure patients over the age of 60 in the Auvergne region, by sharing information via computerized medical records. Only one sensor is provided to the patient: a scale with a remote transmitter. Nurses are provided with a smartphone to access the patient's medical record, as laboratories and pharmacists access it, with the clinical progression (edemas, heart rate, and arterial pressure). The biological monitoring is automatically included in the patient record by the testing labs, and the dispensing pharmacist records the therapy upon each delivery of the prescription medication. An evaluation was made at the end of the first two-year period of the program (355 days of monitoring on average) on the first 558 patients enrolled in the study. The death rate was 12% per year and rehospitalizations (for acute heart failure) were reduced to 13.6% per year (compared with 26 to 40% in European data sets). Alarms and alerts were integrated with priority: a gain or loss of 2 kg set off an alert, while a gain or loss of 5 kg triggered an alarm. If a patient failed to weigh him/herself three days in a row, this too triggered an alert. In the first two years of the Cardiauvergne program, 558 patients were included, 165 of them female, with an average age of 69 years (range: 22-94), and 171 patients (30.6%) were over the age of 80. 67 patients died in those two years, at an average age of 78.6 years (range: 47-94) and 54 patients needed to be rehospitalized one or more times for a new spurt of heart failure, with an average hospital stay of 9.2 days [17].

*To Sum Up. *Most projects and trials rely on the standard connected tools for monitoring HF, such as blood pressure meters, heart rate monitors, weighing scales, and pulse oximeters, which relay the collected information via* Bluetooth*,* 3G *or* 4G*. These projects incorporate the following: self-administered medical questionnaires or forms (symptoms and signs of HF); tools for medical education, particularly disease self-appropriation, food hygiene, and physical activity; tools for patient motivation; tools for therapeutic and hygiene observance; tools for interaction between the patient and healthcare professionals like telephone support centers, tablets, and websites. Thus, several projects provide questionnaires or forms about HF symptoms and signs, such as dyspnea, palpitation, edema, and fatigue corresponding to the acronym EPOF, namely, “*Essoufflement*,* Prise de Poids*,* Oedèmes*, and* Fatigue”*, experienced by the patient. Several projects also include ECG monitoring, and even a video-call.

The main telemedicine 2.0 projects presently undergoing development in France are as follows:

- SCAD project: “*Suivi Cardiologique à Distance*” [remote cardiological follow-up], first initiated in 2005, deployed in the low Normandie, between 2009 April and May 2012, developed by Caen University Hospital
- PIMPS project: “*Plateforme Interactive Médecins Patients Santé*” [doctor-patient interactive health platform], initiated in 2013, developed by* René-Dubos *hospital in Pontoise
- OSICAT project: “*Optimisation de la Surveillance Ambulatoire des Insuffisants Cardiaques par Télécardiologie*” [optimization of outpatient monitoring in HF patients using telecardiology], initiated in 2012, involving 12 local investigation centers coordinated by Toulouse University Hospital
- MEDICA project: “*Monitorage Electronique à Domicile de l'Insuffisance Cardiaque Chronique*” [electronic home monitoring of CHF], initiated in 2014, developed by the* Reunica domicile *and* GMC-solutions santé *groups working in social protection of the elderly
- E-care project: “*Détection des Situations à Risque de Decompensation Cardiaque chez les Patients Insuffisants Cardiaques de Stade III de la NYHA*” [detection of risk situations for cardiac decompensation in HF patients with NYHA Stage-III disease], initiated in 2014, whose medical aspects were developed by Strasbourg University Hospital
- PRADO-INCADO project: This project is scheduled to experiment the E-care platform for at-home monitoring of CHF patients (being deployed in the Strasbourg region). It is run by a group bringing together the Strasbourg University Hospital (*Hôpitaux Universitaires de Strasbourg*), East Regional Health Agency (*Agence Régionale de Santé du Grand Est*), Bas-Rhin branch of France's National Health Insurance (*Caisse Primaire d'Assurance du Bas-Rhin*), and* PREDIMED Technology *start-up. This project likely allows an in-depth study to be carried out designed to improve diagnosis by machine learning and detect abnormalities in CHF patients at an early time point.

## 4. Telemedicine and Arrhythmias in the Elderly Subject

Dary P et al. conducted a study on the telemonitoring of atrial fibrillation. The monitoring was ambulatory in nature, through the daily use of a monitor for an average period of 11 days, 11 hours per day, with detection of arrhythmia and automatic ECG transmission. This monitoring took place daily from 8 to 12 o'clock, alternating day/night periods. This monitor was programmed to conduct an electrocardiogram every hour for better observation. On average, 266 readings were taken per patient, for a total of over 51,000, all patients combined. In all, 200 patients had been enrolled from the start of the study: 45% male and 55% female, with an average age of 67 years. 16% of the enrolled patients were over the age of 80. 35% had a history of treated arrhythmias. A distinction was made between two groups. The first group was in sinus rhythm upon enrollment in the study, with the discovery of an atrial fibrillation in 31% of them and regular episodes of tachycardia in 24%. The second group was already in arrhythmia, with a choice between monitoring heart rate (62%) and rhythm (38%). For the 200 patients enrolled in the study, 63 had a known arrhythmia and 137 patients were in sinus rhythm as of the start of the study. Out of the latter group of 137 patients, 61 had a normal pattern, 43 subjects (22%) had a detected atrial fibrillation, and 33 patients (16%) had episodes of tachycardia. For the 63 patients in arrhythmia, 24 patients had their rhythm monitored, while 39 patients' heart rate was monitored. Thus in this study, for 33% of the patients, the telemonitoring improved the diagnosis and treatment of atrial fibrillation, allowing the therapy to be adjusted and secured according to the rhythm, rate, and conduction time [18].

As for telemedicine in cardiology, the telemonitoring of implanted cardiac rhythm devices (pacemakers and defibrillators) is a developing field in France. Thus, the ECOST study, coordinated by Salem Kacet [19], and Philippe Mabo's EVATEL study [20] are the first two studies conducted in this area. The EVATEL study has not led to any publications, but its results were disappointing overall. The ECOST study shows that the telemonitoring of implantable defibrillators can have a medico-economic impact, with a reduction of inappropriate shocks due to early regulations and a reduction of the hospitalizations related to these shocks. The average savings produced amount to 315 euros per patient per year, by lowering outpatient costs and follow-up costs (baseline consultations and transport), but the program had no effect on hospital costs.

As the TRUST study shows, the telemonitoring of cardiac defibrillators also allows for a reduction in the frequency of face-to-face monitoring without any impact on major undesirable events such as death or stroke [21].

The ECOST and EVATEL studies, conducted in France on 433 and 1501 patients, respectively, confirmed the safety of telemonitoring of defibrillators on “hard” composite criteria, including mortality, cardiovascular hospitalizations, and system malfunction. In addition, telemonitoring made it possible to reduce the incidence of inappropriate shocks by 52% in ECOST and by 37% in EVATEL, with the potential for additional improvement to the longevity of the devices (assessment of the ECOST study).

We should mention that COMPAS (COMPArative Follow-up Schedule with Home Monitoring), with the Biotronik company, involved the monitoring of pacemakers with 538 patients randomized into two arms (remote transmission vs. traditional monitoring). It demonstrated the safety of monitoring patients via telecardiology over a period of 18 months. The COMPAS study, conducted in France, came to the same conclusions for the monitoring of pacemakers, with a 56% reduction in follow-up consultations, also linked to a reduction in hospitalizations for atrial arrhythmia or stroke [22].

Finally, the SETAM (Strategy of Early Detection and Active Management of Supraventricular Arrhythmia with Telecardiology) study is a randomized study involving telemedicine in rhythmology, with two groups of patients, both of which had pacemakers. It demonstrated that telemedicine promoted the early detection of events, with a 66% reduction in hospitalizations related to atrial arrhythmias and the prevention of strokes. The study showed that telecardiology allowed for the earlier diagnosis and treatment of patients with atrial arrhythmias and to reduce the atrial fibrillation burden after nine months of monitoring in patients in sinus rhythm upon enrollment with a score of CHA2DS2-VASc>= 2. 595 patients were enrolled in 57 general hospitals (CHGs) in France, for an average period of 12.8 +/- 3.3 months and an average age of =79 +/- 8 years [23].

*To Sum Up. *Telemedicine and arrhythmias in the elderly subject are not as developed as studies about heart failure in the elderly. But, their attention is only growing year by year with more and more studies concerning the detection and monitoring of supraventricular rhythm disorders as in the SETAM study or the monitoring of pacemaker functionality in older subjects, as in the COMPASS study.

## 5. Perspectives regarding New Developments in Telemedicine

The challenge for “tomorrow” telemedicine is to develop new telemedicine solutions or projects, including and resolving several medical problems and difficulties, such as

- The specificities (no appetite for new technologies and new uses) and problems (e.g., falls, malnutrition, mild cognitive impairment, etc.) of elderly subjects, who are the main subjects affected by chronic diseases
- The coexistence of several chronic pathologies (e.g., CHF, DM, chronic obstructive pulmonary disease [COPD], etc.) and comorbidities (arterial hypertension, renal failure, etc.) in the same individual, while providing comprehensive and “global” care for the individual patient in all its medical and societal dimensions
- The multiplicity of care structures and medical organizations (e.g., with or without human resources, telemedical center, etc.)
- The logistical barriers to implementing telehealth proving to be significant, as many health systems are not yet designed for these technologies to be integrated within existing information systems

In the chronic disease setting, new remote sensors and tailored questionnaires are presently being integrated into telemedicine platform, including remote glucose meters, actimeters, and electronic spirometers, along with new knowledge in the form of ontologies in order to enhance the telemedicine platform and broaden its utility to other chronic diseases like Diabetes Mellitus (DM) and COPD. In this setting, additional personnel and specific protocols are necessary that must be specific for each chronic disease and targeted for each patient, while integrating the possibility for each patient to exhibit more than one chronic disease. Most of these protocols must still be funded by means of existing resources or external grants.

These diseases share a number of points with HF in terms of epidemiology and natural history. Like HF, DM and COPD are among the most common diseases in developed countries and thus represent a major public health concern for our societies. Crucially, like HF, they are accompanied by frequent hospital admissions and readmissions for well-known causes. These causal factors can be detected, enabling professionals to act ahead of time, as in CHF, thereby avoiding disease progression. Developing warning alerts for these chronic diseases should enrich the existing system.

## 6. Conclusion

Since the beginning of the 2000s, several telemedicine projects and trials focused on heart failure have been developed. Numerous telemedicine projects based on connected objects or new information and communication technologies (ICT) (telemedicine 2.0) have emerged over the last 5 years or are still under development as for computer science heart failure. Their potential contribution in terms of mortality, morbidity, and number of hospitalizations avoided is currently under study. Their impact in terms of health economics is likewise being investigated, taking into account the fact that the economic and social benefits brought up by telemedicine solutions had previously been validated by the first-born telemedicine projects. For example, in the SCAD project, 90 patients were randomized from April 2009 to May 2011. The population was elderly, with a mean age of 78 ± 6 years, mostly male (78%), and at high risk of rehospitalization (mean brain natriuretic peptide [BNP] level of 1,025 ± 950pg/mL). At 12 months, 1040 days of hospitalization for acute HF were recorded. Monitoring by educational telemedicine significantly reduced the number of hospital days for acute HF: 590 days in the control group vs. 450 days in the telemedicine group (*p=*0.044). The criterion “*death or hospitalization for acute HF*” occurred less frequently in the telemedicine group, namely, 57.8% in the control group vs. 35.6% in the telemedicine group (*p *<0.05). During HF readmissions, telemedicine-treated patients exhibited lower intrahospital mortality: 18.2% vs. 0% (*p *<0.02).

Numerous noninvasive telemonitoring projects have been conducted in the CHF area; relatively few have been run within the “telemedicine 2.0” setting, using both ICT and the web. Although the earliest telemedicine projects confirmed certain clinical benefits, they mostly demonstrated its economic benefits. The Tele-HF2 study is the first to well document the interest of the telemedicine in the CHF field, resulting in clinically relevant outcomes with statistical significance (percentage of days lost due to unplanned cardiovascular hospital admissions and all-cause death).
